# E-cigarette Use, or Vaping, Practices and Characteristics Among Persons with Associated Lung Injury — Utah, April–October 2019

**DOI:** 10.15585/mmwr.mm6842e1

**Published:** 2019-10-25

**Authors:** Nathaniel Lewis, Keegan McCaffrey, Kylie Sage, Chia-Jung Cheng, Jordan Green, Leah Goldstein, Hillary Campbell, Deanna Ferrell, Nathan Malan, Nathan LaCross, Alejandra Maldonado, Amy Board, Arianna Hanchey, Dixie Harris, Sean Callahan, Scott Aberegg, Ilene Risk, Sarah Willardson, Amy Carter, Allyn Nakashima, Janae Duncan, Cindy Burnett, Robyn Atkinson-Dunn, Angela Dunn

**Affiliations:** ^1^Epidemic Intelligence Service, CDC; ^2^Utah Department of Health; ^3^National Center for Environmental Health, CDC; ^4^Intermountain Healthcare, Salt Lake City, Utah; ^5^University of Utah Health, Salt Lake City, Utah; ^6^Salt Lake County Health Department, Salt Lake City, Utah; ^7^Davis County Health Department, Clearfield, Utah; ^8^Weber-Morgan Health Department, Ogden, Utah.

In August 2019, the Utah Department of Health (UDOH) received reports from health care providers of several cases of lung injury in persons who reported use of electronic cigarette (e-cigarette), or vaping, products (*1*,*2*). To describe the characteristics of medical care, potentially related conditions, and exposures among 83 patients in Utah, detailed medical abstractions were completed for 79 (95%) patients. Among patients receiving chart abstractions, 70 (89%) were hospitalized, 39 (49%) required breathing assistance, and many reported preexisting respiratory and mental health conditions. Interviews were conducted by telephone or in person with 53 (64%) patients or their proxies, and product samples from eight (15%) of the interviewed patients or proxies were tested. Among 53 interviewed patients, all of whom reported using e-cigarette, or vaping, products within 3 months of acute lung injury, 49 (92%) reported using any products containing tetrohydrocannabinol (THC), the principal psychoactive component of cannabis; 35 (66%) reported using any nicotine-containing products, and 32 (60%) reported using both. As reported in Wisconsin and Illinois (*1*), most THC-containing products were acquired from informal sources such as friends or illicit in-person and online dealers. THC-containing products were most commonly used one to five times per day, whereas nicotine-containing products were most commonly used >25 times per day. Product sample testing at the Utah Public Health Laboratory (UPHL) showed evidence of vitamin E acetate in 17 of 20 (89%) THC-containing cartridges, which were provided by six of 53 interviewed patients. The cause or causes of this outbreak is currently unknown (*2*); however, the predominant use among patients of e-cigarette, or vaping, products with prefilled THC-containing cartridges suggests that the substances in these products or the way in which they are heated and aerosolized play an important role in the outbreak. At present, persons should not use e-cigarette, or vaping, products that contain THC. In addition, because the specific cause or causes of lung injury are not yet known and while the investigation continues, persons should consider refraining from use of all e-cigarette, or vaping, products.

During August–October 2019, possible cases of e-cigarette, or vaping, product use–associated lung injury (EVALI) in Utah were investigated to determine symptoms, medical care history, and exposures related to the injury. Cases were classified as confirmed or probable according to established case definitions (*3*). Medical record abstraction was completed using a detailed form provided by CDC in September 2019. Interviews were conducted with patients, or a proxy (a spouse or parent), using an adaptation of a questionnaire developed in Illinois and Wisconsin in consultation with CDC during investigation of cases in those states (*1*). Medical record abstractions were conducted by UDOH staff members. Interviews were conducted by UDOH staff members or local health department staff members in-person or by telephone to assess product acquisition and use behaviors.

UDOH and Utah local health departments collected e-cigarette, or vaping, products from patients for testing using gas chromatography–mass spectrometry at UPHL to identify peaks for known chemical substances (including nicotine and THC) through nontargeted testing followed by partial verification of results with targeted tests for analytes that have known chemical standards (nicotine and vitamin E acetate, along with 16 others*) or known m/z values (i.e., mass) and relative retention times (myclobutanil and thiodiglycol) (*4*).

During August 6–October 15, 2019, 83 confirmed and probable cases of EVALI were reported, primarily by clinicians and Utah Poison Control Center, to UDOH. The overall prevalence was 26 per 1,000,000 population. Most (86%) of the patients lived in Salt Lake County and surrounding urban counties (Davis, Morgan, Weber, and Utah); 14% lived in outlying counties. Abstraction of medical records was completed for 79 (95%) patients, and 53 (64%) interviews were completed.

Among the 83 patients, 69 (83%) were male, and the median age was 26 years (range = 14–66 years) (Table 1). Among the 79 patients for whom medical record data were available, 70 (89%) were hospitalized during June 5–September 23 (median duration = 4 days; range = 1–17 days), including 35 (44%) who required intensive care unit (ICU) admission; nine (11%) were not hospitalized. Many patients required respiratory support; continuous or bilevel positive airway pressure was required by 30 (38%), and endotracheal intubation and mechanical ventilation was required by nine (11%). Fifty-nine (75%) patients were treated with steroids. Twenty (25%) patients received a diagnosis of acute respiratory distress syndrome. Patients reported having histories of asthma, 16 (20%); anxiety, 27 (34%); depression, 18 (23%); hypertension, four (5%); and heart failure, one (1%). Approximately half of the patients had at least one of these preexisiting conditions. Patients also reported smoking combustible marijuana (43%), tobacco (54%), or both (24%).

**TABLE 1:** Characteristics of patients with electronic cigarette (e-cigarette), or vaping, product use–associated lung injury, (N = 83) — Utah, April–October 2019

Characteristic (no. with available information)	No. (%)
**Sex (83)**	
Male	69 (83)
Female	14 (17)
**Age group (yrs) (83)**	
14–19	11 (13)
20–29	43 (52)
30–39	23 (28)
40–66	6 (7)
Required medical care/In-care diagnoses* (79)	
Hospitalization	70 (89)
ICU admission	35 (44)
CPAP/BiPAP support (No intubation)	30 (38)
Intubation and mechanical ventilation	9 (11)
Treated with steroids	59 (75)
Acute respiratory distress syndrome	20 (25)
Preexisting conditions* (79)	
Asthma	16 (20)
Chronic obstructive pulmonary disease	2 (3)
Anxiety	27 (34)
Depression	18 (25)
Hypertension	4 (5)
Heart failure	1 (1)
One or more of the above	42 (53)
Smoking history*^,†^ (79)	
Marijuana	34 (43)
Tobacco	43 (54)
Both marijuana and tobacco	19 (24)

**Abbreviations:** BiPAP = bilevel positive airway pressure; CPAP = continuous positive airway pressure; ICU = intensive care unit.

* Denominators based on total patients with medical abstraction data available (unknowns included in denominator).

^†^ Includes current and former smokers.

Among the 53 patients interviewed, 49 (92%) reported use of THC-containing e-cigarette, or vaping, products during the 3 months preceding illness (Table 2); 35 (66%) reported using nicotine-containing products; and 32 (60%) reported using both THC- and nicotine-containing products. Seventeen (32%) patients reported exclusive use of THC-containing products, whereas three (6%) reported exclusive use of nicotine-containing products. Use of three brands of prefilled THC-containing cartridges was reported frequently by patients; these included Dank Vapes (21, 40%), Rove (19, 36%), and Golden Gorilla (11, 21%). Seventeen (32%) patients reported using more than one of these brands.

**TABLE 2:** Self-reported product use behaviors in the 3 months before injury onset in interviewed patients with electronic cigarette (e-cigarette), or vaping, product use–associated lung injury (N = 53) — Utah, April–October 2019

Product use and behavior	No. (%)
**THC-containing product use**	
Any use	49 (92)
Exclusive use	17 (32)
**THC-containing cartridge brands used**	
Dank Vapes	21 (40)
Rove	19 (36)
Golden Gorilla	11 (21)
Two or more of the above	17 (32)
**Nicotine-containing product use**	
Any use	35 (66)
Exclusive use	3 (6)
**Both THC- and nicotine-containing product use**	32 (60)

**Abbreviation:** THC = tetrahydrocannabinol.

Patients reported a total of 131 e-cigarette, or vaping, products used during the 3 months before illness and for which the method of acquisition was known; 84 of these were THC-containing products, and 47 were nicotine-containing products (Table 3). Most THC-containing products were acquired through informal sources, including friends (44%), in-person dealers (25%), and online dealers (24%). Five products were purchased at an out-of-state dispensary and one at an in-state vape shop selling these products illicitly. Among 84 THC-containing products used, frequency of use was reported for 70 of 84 (83%). Approximately two thirds (65%) of the THC-containing products were used ≤5 times per day. Among 47 nicotine-containing products used, frequency of use was reported for 29 of 47 (62%). The majority of the nicotine-containing products were used >25 times per day (55%) and were acquired primarily through in-state vape shops (49%) or convenience stores and gas stations (18%).

**TABLE 3:** Characteristics of tetrahydrocannabinol (THC)- or nicotine-containing products used in the 3 months preceding illness onset in patients with electronic cigarette (e-cigarette), or vaping, product use–associated lung injury (N = 131) — Utah, April–October 2019

Characteristic	No. (%)	
	THC-containing products (N = 84)	Nicotine-containing products (N = 47)
**Method of acquisition**		
Friend	37/84 (44)	9/47 (19)
Dealer	21/84 (25)	0/47 (0)
Online dealer	20/84 (24)	7/47 (15)
Out-of-state dispensary	5/84 (6)	1/47 (2)
In-state vape shop	1/84 (1)	23/47 (49)
Convenience store/gas station	0/84 (0)	7/47 (18)
**Frequency of use (times per day)**		
<1	8/70 (11)	3/29 (10)
1–5	38/70 (54)	5/29 (17)
6–25	7/70 (10)	5/29 (17)
>25	17/70 (24)	16/29 (55)
**Testing**		
Products tested at UPHL*	19/84 (23)	20/47 (43)
Products found to contain THC	19/19 (100)	0/20 (0)
Products found to contain nicotine	1/19 (5)	20/20 (100)
Products found to contain vitamin E acetate	17/19 (89)	0/20 (0)

**Abbreviation:** UPHL = Utah Public Health Laboratory.

* THC-containing cartridges tested came from six patients and nicotine-containing vaping liquids came from eight patients. Test results might therefore represent clusters of purchase or use by these patients rather than fully independent samples.

To date, UDOH and Utah local health departments have collected 72 products from eight (15%) of 53 patients interviewed. Products tested at UPHL comprised 19 prefilled THC-containing cartridges from six patients and 20 nicotine-containing vaping liquids (19 bottled e-liquids and one from an atomizer) from six patients; six patients provided both THC- and nicotine-containing samples, and two provided only nicotine-containing samples). Among the 19 THC-containing cartridges, THC was detected in 19 of 19 (100%), nicotine was detected in one (5%), and evidence of vitamin E acetate was detected in 17 (89%). Samples of nicotine-containing e-liquid, in contrast, only showed evidence of nicotine and no evidence of THC or vitamin E acetate. No other analytes were found.

## Discussion

In this study of 83 Utah residents with EVALI during August–October 2019, approximately 90% of patients were hospitalized, approximately half in ICUs, and more than half of hospitalized patients required some form of respiratory support. Three quarters were treated with steroids. It is not known why some patients have more severe illness; preexisting behaviors and conditions might play a role in injury exposure, onset, and injury progression. Whereas some patients reported preexisting respiratory problems, most were previously in good physical health, although many reported that they self-identified as current or former smokers of combustible marijuana or tobacco. Many patients reported histories of anxiety or depression, which might influence the use or patterns of e-cigarette, or vaping, product use, particularly products containing THC (*5*).

The median age of patients in this study was 26 years, 3 years older than the national median of 23 years; more than one third were aged ≥30 years. The older age profile in Utah suggests a need to focus on adult populations at risk in addition to younger persons. Utah’s rate of adult e-cigarette use (5.1%) was similar to the national rate (4.6%) in 2017 (the most recent year for which state and national data are available), and e-cigarette use among youths (7.6%) was lower than the national rate (13.2%) in 2017, although rates in all states increased in 2018 and 2019 (*6*). As of October 15, 2019, Utah’s rate of EVALI was 26 per 1 million compared with four per 1 million nationally (*7*). More research is needed to identify the constellation of risk factors influencing the high rate of EVALI in Utah.

Most patients in this analysis reported using THC-containing products (which are illegal for nonmedical use in Utah) that were sold as prefilled cartridges and obtained from informal sources. Compared with Illinois, Wisconsin, and nationally, patient use rates for prefilled THC-containing cartridges in Utah were even higher while those for nicotine-containing products were lower, reinforcing the finding that unregulated THC-containing cartridges play an important role in this outbreak (*1*,*2*). Products labeled with three different brand names, Dank Vapes, Rove, and Golden Gorilla, were each reported by a substantial proportion of patients (20%–40%), although packaging for these brands can be reproduced or purchased online. In Illinois and Wisconsin, Dank Vapes was reported far more than any other brand, Rove was reported by a few patients, and Golden Gorilla was not reported at all (*1*,*2*). Although the respective market shares of these brands are unknown, findings from the Utah investigation might reflect a distinct pattern of illicit THC supply and production in Utah or the western United States compared with that in the Midwest and other areas of the United States.

Vitamin E acetate was identified in the majority of THC cartridge samples tested at UPHL; however, these samples only represent six patients. National data summarized recently in a news report suggested that vitamin E acetate is a now common diluent in THC cartridges (*8*). Quantification of vitamin E acetate in Utah’s samples is pending; however, testing of other case samples by the Food and Drug Administration and other laboratories has shown vitamin E acetate concentrations of 31%–88% and lower-than-expected THC concentrations (14%–76% versus the typically advertised 75%–95%) (*8*). The potential role of vitamin E acetate in lung injury remains unknown; however, the identification of vitamin E acetate among products collected from patients in Utah and elsewhere indicates that the outbreak might be associated with cutting agents or adulterants (*9*). Ascertaining the potential contribution of diluents to the current outbreak will require data from multiple states and analysis at the national level.

The findings in this report are subject to at least five limitations. First, because interviews were not conducted with 30 (36%) patients, nonresponse could introduce selection bias and result in inaccurate estimation of specific substances used and use patterns. Second, because nonmedical THC use currently is illegal in Utah, self-reported use could be influenced by the perceived stigma of illicit substance use or fear of legal repercussions, which might result in underreporting of use. Third, case reporting in Utah relies on clinician reports, which, to date, have come largely from pulmonologists and critical care physicians. Consequently, there is possible reporting bias toward hospitalized patients and those with more severe respiratory symptoms. Fourth, care requirements or preexisting conditions are not always reported on medical charts, meaning that rates could be higher than reported. Finally, because laboratory analysis and coordination are currently limited, there might be factors contributing to the lung injury not yet identified.

Effective interventions to halt this outbreak might require a stronger partnership between public health and law enforcement agencies to identify the locations of supply and distribution chains that are contributing to lung injuries, alongside targeted messaging to consumers. UDOH has initiated a print and social media campaign to alert the public to the potential dangers associated with use of THC-containing e-cigarette, or vaping, products. At present, persons should not use e-cigarette, or vaping, products that contain THC. In addition, because the specific cause or causes of lung injury are not yet known and while the investigation continues, persons should consider refraining from use of all e-cigarette, or vaping, products (*10*).

**Summary**

**What is already known about this topic?**

An outbreak of e-cigarette, or vaping, product use–associated lung injury (EVALI) of unknown source is ongoing in the United States.

**What is added by this report?**

Medical abstractions were completed for 79 Utah patients, 53 of whom were interviewed. Almost all patients reported using tetrahydrocannabinol (THC)-containing vaping cartridges. Most patients were hospitalized, half required breathing assistance, many reported preexisting respiratory and mental health conditions, and many identified as current or former smokers of combustible marijuana or tobacco. Most THC-containing products, acquired from six patients and, tested at Utah Public Health Laboratory, contained vitamin E acetate.

**What are the implications for public health practice?**

At present, persons should not use e-cigarette, or vaping, products containing THC. In addition, because the specific cause or causes of lung injury are not yet known and while the investigation continues, persons should consider refraining from use of all e-cigarette, or vaping, products.

## Footnotes

* The other 16 analytes are diazion, phorate, terbuphos, tetramine, paraoxon parathion, pentazocine, scopolamine, codeine, strychnine, aldrin, endrin, dichlorodiphenyltrichloroethane (DDT), fentanyl, dichlorodiphenyldichloroethylene (DDE), arecoline, pilocarpine, and morphine.
